# *Epigynum auritum*-Derived Near-Infrared Carbon Dots for Bioimaging and Antimicrobial Applications

**DOI:** 10.3390/molecules30020422

**Published:** 2025-01-20

**Authors:** Wenfeng Shi, Jiahui Li, Junmei Pu, Guiguang Cheng, Yaping Liu, Shanshan Xiao, Jianxin Cao

**Affiliations:** 1 Faculty of Food Science and Engineering, Kunming University of Science and Technology, Kunming 650500, China; wfshi@stu.kust.edu.cn (W.S.); 20222225041@stu.kust.edu.cn (J.L.); 20222125014@stu.kust.edu.cn (J.P.); ggcheng@kust.edu.cn (G.C.); liuyaping@kust.edu.cn (Y.L.); 2 Yunnan International Joint Laboratory of Green Food Processing, Kunming 650500, China

**Keywords:** near-infrared carbon dots, bacteriostatic, bioimaging

## Abstract

The use of biomass feedstocks for producing high-value-added chemicals is gaining significant attention in the academic community. In this study, near-infrared carbon dots (NIR-CDs) with antimicrobial and bioimaging functions were prepared from *Epigynum auritum* branches and leaves using a novel green synthesis approach. The spectral properties of the synthesized NIR-CDs were characterized by ultraviolet–visible (UV-Vis) absorption and fluorescence spectroscopy. The crystal structures of the NIR-CDs were further characterized by high-resolution transmission electron microscopy (HR-TEM), X-ray photoelectron spectroscopy (XPS), Fourier transform infrared spectroscopy (FT-IR), nuclear magnetic resonance (NMR), and X-ray diffraction (XRD). The NIR-CDs exhibited minimal toxicity, excellent biocompatibility, and high penetrability in both in vivo and in vitro environments, making them ideal luminescent probes for bioimaging applications. Moreover, the antimicrobial activity of NIR-CDs was tested against *Staphylococcus aureus* (*S. aureus*) and *Escherichia coli* (*E. coli*), showing significant bacterial growth inhibition. The antimicrobial effect is likely attributed to the NIR-CDs disrupting the cell membrane integrity, leading to the leakage of the intracellular contents. Therefore, NIR-CDs hold promise as fluorescent bioimaging probes and antimicrobial agents.

## 1. Introduction

In recent years, carbon dots (CDs) have gained significant attention in nanoscience and technology as an emerging nanomaterial [1,2]. Compared to traditional semiconductor quantum dots (QDs) and organic fluorescent dyes, CDs offer distinctive optical properties, excellent biocompatibility, favorable colloidal stability, a low cost, and easy surface functionalization [3]. These advantages have rendered CDs highly attractive for applications in a plethora of applications, including sensors, drug delivery, phototherapy, energy storage systems, biosensors, and antibacterial agents [4,5,6]. A review of the literature reveals that the majority of reported CDs emit light in the blue or green wavelength regions, which can have detrimental effects on living cells or biological systems [7,8]. This presents a significant challenge for the practical application of CDs in bioimaging [9]. It has been demonstrated that fluorescence emission in the near-infrared (NIR) region can facilitate higher imaging resolution and contrast, due to the reduction in autofluorescence and the enhancement of the tissue penetration depth [10,11,12]. It is therefore of significant importance and urgency to develop near-infrared carbon dots (NIR-CDs). To date, a number of research groups have successfully synthesized CDs that emit in the red and NIR regions [13,14,15]. For example, Li et al. synthesized CDs (680 nm) from spinach through a solvothermal process. Yang’s group developed two types of NIR-emitting CDs using o-phenyl-enediamine as a precursor [16,17]. However, these CDs have certain limitations, including the use of toxic carbon sources, weak fluorescence emission in the NIR region, and high costs, which restrict their practical application. Recently, Xu’s group developed a simple method using magnolia leaves as a carbon source to synthesize CDs with deep-red emission and narrow full-width at half-maximum (FWHM) [6,18]. However, research on NIR-CDs derived from natural resources as fluorescent probes for in vivo imaging and with antibacterial effects is still in its early stages [6,19].

*Epigynum auritum*, a member of the Apocynaceae family, is native to southern Yunnan, including regions such as Xishuangbanna and Jinghong. This plant is recognized for its heat-clearing, detoxifying, and pain-relieving properties. The Dai ethnic group has traditionally used its bark and roots for medicinal purposes, with documented folk medicinal uses [20]. Due to the complex and time-consuming nature of isolating active compounds from plants, we successfully prepared NIR-CDs using the branches and leaves of *Epigynum auritum* as precursors via a one-step solvothermal process in this study [21]. This method not only efficiently utilizes plant resources but also broadens the applications of carbon dots in bioimaging and antibacterial fields, representing a significant advancement in the preparation and practical applications of carbon dots [22,23]. The synthesized NIR-CDs exhibit a maximum emission peak at 678 nm, with a broad excitation range extending from ultraviolet to red light, and show no excitation dependency. Under 409 nm excitation, the quantum yield of the NIR-CDs reached 27.22%. Leveraging their fluorescence and structural properties, we conducted studies on their imaging capabilities in vivo and in vitro, as well as their antibacterial potential. The results show that these NIR-CDs have good biocompatibility, extended retention time, and minimal residue, indicating their excellent performance as near-infrared fluorescent probes [24]. In addition, the NIR-CDs effectively inhibited the growth of *Staphylococcus aureus* (*S. aureus*) and *Escherichia coli* (*E. coli*), demonstrating significant antibacterial properties. This provides valuable insights for the development of low-cost fluorescent probes and antibacterial agents [25,26].

## 2. Results

### 2.1. Characterization of NIR-CDs

**Figure 1:**
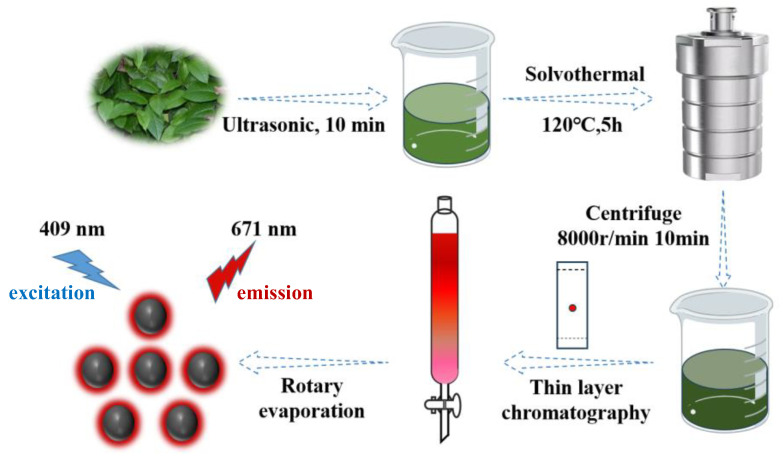
Preparation process of near-infrared carbon dots (NIR-CDs).

First, 1 g of dried *Epigynum auritum* branches and leaves was subjected to acetone (50 mL) heat treatment at 120 °C for 5 h. The product was then purified by silica column chromatography to yield NIR-CDs (Figure 1). The resulting NIR-CD solution was concentrated using a rotary evaporator (GeneVac, Cambridge, UK) and dried in a vacuum oven to yield a dark green solid. The dark green solid had a mass of 10 milligrams.

**Figure 2:**
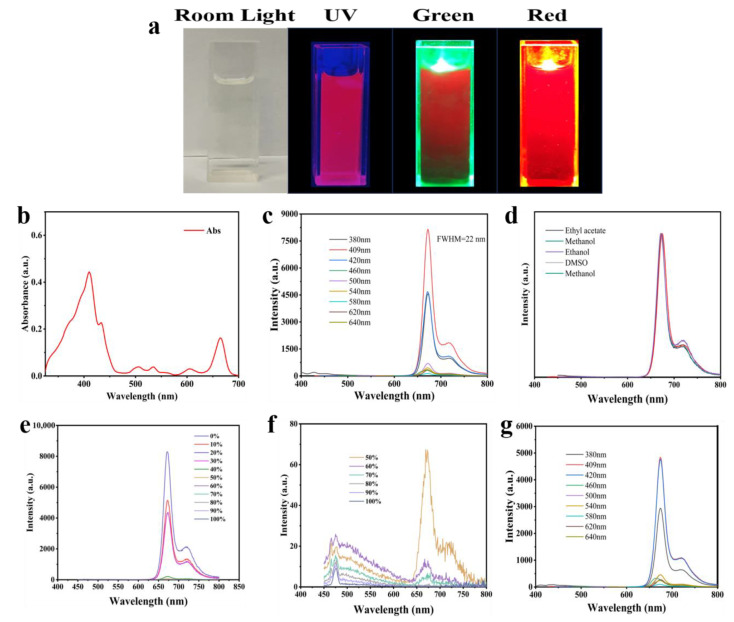
(**a**) Photos of NIR-CD solutions under different irradiation lights. (**b**) Absorption spectra; (**c**) FL emission spectra of NIR-CDs in acetone solution. (**d**) FL emission spectra of NIR-CDs in different solvents. (**e**,**f**) FL emission spectra of NIR-CDs in ethanol solution at different water contents. (**g**) FL emission spectra of NIR-CDs in DMSO solution at different excitation wavelengths.

Bright red light was emitted when the NIR-CDs were dissolved in acetone and excited with light of different wavelengths (Figure 2a). This is attributed to the broad absorption band of the NIR-CD solution, spanning from UV to deep red light. The maximum ultraviolet–visible (UV-Vis) absorption peak of the NIR-CD solution occurred at approximately 409 nm (Figure 2b). Figure 2c shows the fluorescence emission profiles of the NIR-CDs in acetone solution under different excitation wavelengths. The results indicate that the optimal emission peak of the NIR-CDs was at 671 nm with an FWHM of 22 nm. The absolute quantum yield (QY) of the NIR-CDs in acetone solution at the optimal excitation wavelength (409 nm) was 27.22%, with an average fluorescence lifetime of 5.799 ns (see Figures S1 and S2, Supporting Information), exhibiting biexponential decay. As the solvent polarity increased from ethyl acetate to methanol, the NIR-CDs exhibited a consistent emission peak at 717 ± 2 nm under 409 nm excitation, attributed to vibrational resonance structures (Figure 2d) [27,28,29]. In highly polar solvents, such as water, NIR-CDs tended to aggregate and showed poor solubility. To control the aggregation degree of the NIR-CDs, we measured their fluorescence spectra in various water/ethanol mixtures (Figure 2e). The NIR-CD solutions in pure ethanol showed a pronounced fluorescence intensity, which decreased with increasing water content. This phenomenon is attributed to aggregation-induced quenching (ACQ) effects. At 60 vol% water, the ACQ effect became significant, leading to a marked decrease in the fluorescence intensity (Figure 2f). To explore the application of NIR-CDs in bioimaging, we characterized their optical properties in dimethyl sulfoxide (DMSO) solution. The results indicated that the fluorescence emission spectra of NIR-CDs in DMSO exhibited similar peaks and emission behavior to those in acetone solution (Figure 2g), confirming their suitability for biological imaging applications [30,31].

**Figure 3:**
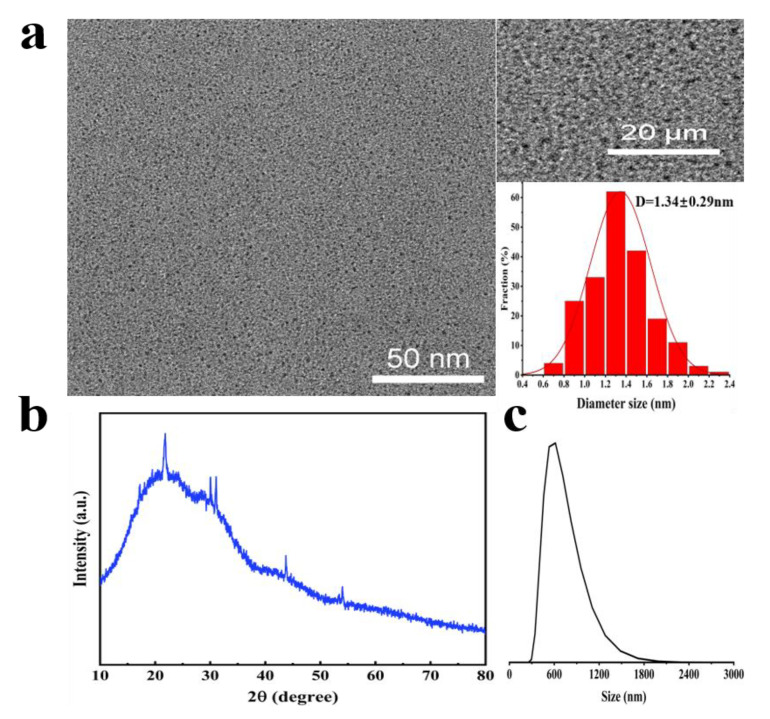
(**a**) TEM and HR-TEM images: the corresponding dot diameter distribution histogram from 200 NIR-CDs; (**b**) XRD pattern; (**c**) DLS analysis of NIR-CDs.

We then characterized the morphology and chemical structure of the NIR-CDs. The NIR-CDs exhibited a quasi-spherical shape and were well dispersed, with no visible cross-linking or agglomeration. The particle sizes ranged from 0.2 nm to 3 nm, with a narrow and symmetrical distribution. The average particle size was approximately 1.34 ± 0.29 nm, determined from the analysis of 200 NIR-CDs quantified from TEM images (Figure 3a). The HRTEM images did not show clear lattice fringes. As shown in Figure 3b, the XRD spectra of the NIR-CDs show a broad diffraction peak near 22° and two shoulder peaks near 30° and 44°, corresponding to the (002), (040), and (100) planes of graphitic carbon. This phenomenon may result from the high degree of disorder in the carbon atoms, indicating the successful synthesis of carbon dots [32,33,34,35]. These results suggest that the prepared NIR-CDs have a disordered carbon structure with significant amounts of amorphous carbon [36]. Additionally, four sharp diffraction peaks were observed: 22 degrees, 33 degrees, 44 degrees, and 54 degrees. This phenomenon may result from cellulose, lignin, proteins, polysaccharides, and impurities [37,38,39,40,41]. The nanoparticle size measured by Dynamic Light Scattering (DLS) is typically larger than that observed via TEM. The DLS measurements indicated that the average size of the NIR-CDs in acetone solution was 551.2 ± 128.3 nm (Figure 3c). The TEM images revealed that the core size of the NIR-CDs remained relatively consistent, while the DLS measurements showed a notable enlargement of the hydrodynamic size, likely due to the presence of polymer chains on the surface of the NIR-CDs and potential particle aggregation in solution.

**Figure 4:**
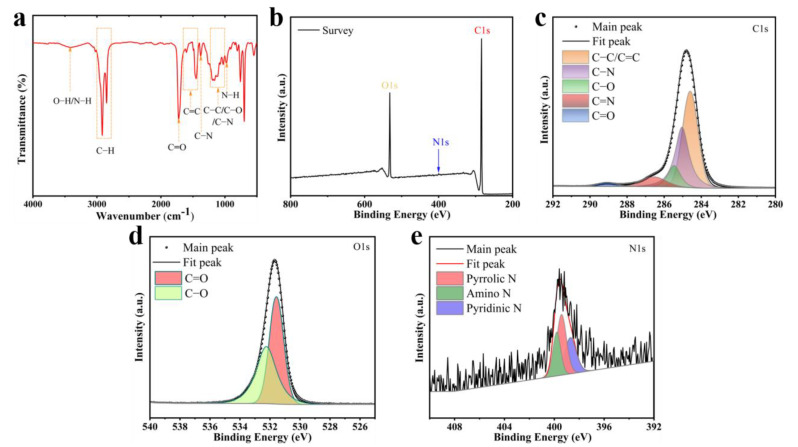
(**a**) FT-IR spectrum; (**b**) XPS survey spectra; (**c**) C1s XPS; (**d**) O1s XPS; and (**e**) N1s spectra of NIR-CDs.

FT-IR and XPS analyses were conducted to determine the elemental composition and surface groups of the NIR-CDs. The FT-IR spectra of the NIR-CDs (Figure 4a) show absorption peaks at 3418 cm^−1^, associated with the stretching vibrations of O-H and N-H bonds. The C-H methyl stretching vibrations are represented by double peaks at 2916 cm^−1^ and 2847 cm^−1^. The absorption peaks at 1495 cm^−1^ and 1380–1450 cm^−1^ correspond to the stretching vibrations of C=C and C-N bonds [42]. The absorption peaks at 1195 cm^−1^ are attributable to the stretching vibrations of the C-C, C-O, and C-H bonds. The absorption peak at 975 cm^−1^ is attributed to the stretching vibration of the N-H bond [43]. The absorption peaks at 700 cm^−1^–750 cm^−1^ are attributed to the stretch–bend vibrations of C-C, C-O, and C-H bonds, respectively [44,45]. These findings suggest that the synthesized NIR-CDs are abundant in carbonyl, hydroxyl, amide, ether linkages, and aromatic ring moieties [46,47]. Figure 4b–e illustrate the XPS characterization results for the NIR-CDs. The complete XPS spectrum (Figure 4b) shows three typical peaks at 284.0 eV, 532.0 eV, and 400.0 eV, indicating that the NIR-CDs are mainly composed of carbon (81.14%), oxygen (17.83%), and nitrogen (1.03%). The C1s spectrum in Figure 4c shows five characteristic peaks attributed to C-C/C=C (284.6 eV), C-N (285.05 eV), C-O (285.5 eV), C=N (286.5 eV), and C=O (289.1 eV). The O1s spectral band in Figure 4d shows characteristic peaks for C=O and C-O at 531.5 eV and 532.2 eV, respectively. The N1s spectrum (Figure 4e) shows three peaks at 398.7 eV, 399.4 eV, and 399.8 eV, corresponding to pyridine N, pyrrole N, and graphite N, respectively [48]. Additionally, the ^1^H NMR spectra (Figure S3) show significant chemical shift peaks for aromatic hydrogen, pyrrole hydrogen, and pyridine, which are consistent with the characterization results of XRD and FTIR [49,50,51]. The TEM, FTIR, and XPS analyses indicate that the red emission of the NIR-CDs is attributed to their larger conjugation system and higher degree of graphitization [51,52].

**Figure 5:**
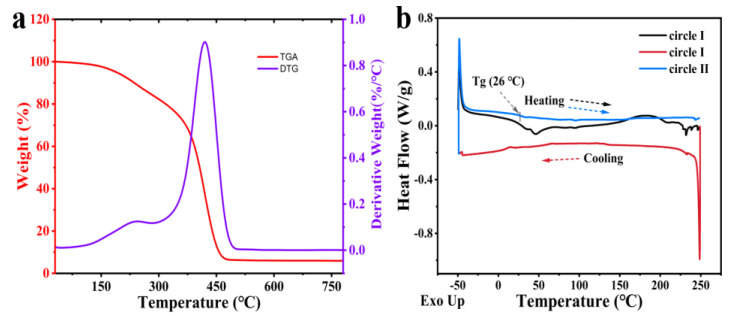
(**a**) TGA (red curve) and DTG (blue curve) thermograms; (**b**) DSC thermograms of NIR-CDs.

We also used Thermogravimetric Analysis (TGA) and Differential Thermogravimetry (DTG) to elucidate the chemical structure of the NIR-CDs. The TGA curves (red line in Figure 5a) show a significant weight loss process for the NIR-CDs, with stabilization at 70 °C, followed by a gradual weight decrease until the residual weight at 465 °C is 5.94%. This result indicates the presence of polymer chains in the NIR-CDs [53]. The DTG curves (blue line in Figure 5a) show the rate of weight loss, characterized by a two-stage degradation. The first degradation occurs at ~250 °C and is attributed to the decomposition of oxygen-containing groups (e.g., ether bonds, unsaturated double bonds, and hydroxyl groups). The second degradation occurs between 300 °C and 500 °C and corresponds mainly to polymer chain breakage and pyrolysis of the internal cross-linked polymer chains [54]. The final residual weight was 5.94% at 500 °C. To further validate the polymer characterization of the NIR-CDs, we investigated their phase transition by performing two heating processes to eliminate interferences (Figure 5b). The NIR-CDs demonstrated a glass transition temperature (Tg) of 26 °C, which rendered the dried NIR-CDs in a viscous colloidal state when exposed to ambient conditions. As the temperature rises, the NIR-CDs undergo a gradual transition to a liquid state, a phenomenon that is corroborated by empirical observations. Therefore, based on the above data and literature reports, the NIR-CDs exhibit unique polymer properties.

### 2.2. Toxicity and Imaging

#### 2.2.1. In Vitro Bioimaging and Toxicity

**Figure 6:**
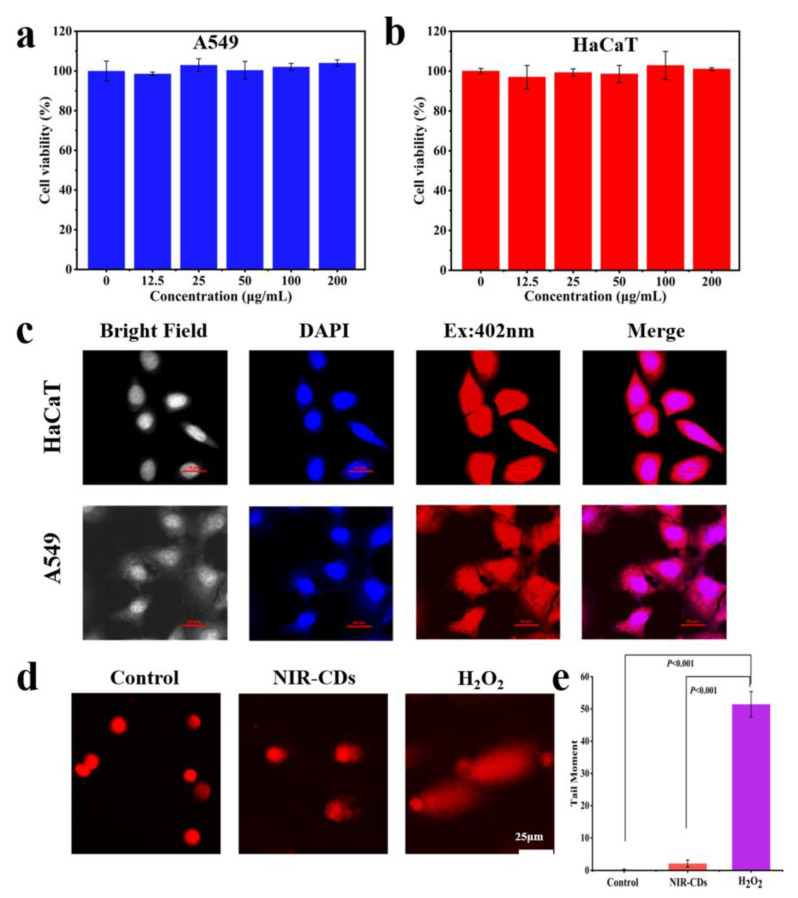
Cell viability of (**a**) A549 and (**b**) HaCaT cells. (**c**) Cellular uptake images of NIR-CDs, Scale bar, 20 μm. (**d**) Representative images of the HaCaT cells treated with PBS or NIR-CDs in the comet assay. Scale bar, 25 μm. (**e**) Analyses of the tail length of the HaCaT cells treated with PBS or NIR-CDs in the comet assay. There is a statistically significant difference between positive control and NIR-CD-treated cells (*p* < 0.001).

The promising properties of the deep red luminescent NIR-CD have prompted us to investigate its potential for use in deep tissue bioimaging. Thus, we conduct extensive toxicity testing using HaCaT cells (human keratinocytes cells) and A549 cells (human lung carcinoma cells) as model systems to ensure their safety and efficacy. First, the human normal cell line HaCaT and the human tumor cell line A549 can be used as an extensive cellular model to evaluate the biocompatibility and toxicity of NIR-CDs. Secondly, these cells can model human skin wounds and lung disease environments, which are pertinent to our research goals in the areas of antimicrobial agents and bioimaging. Firstly, the in vitro safety of the NIR-CDs was assessed using the Cell Counting Kit-8 (CCK-8) assay. It is noteworthy that no significant differences were observed between the treatment and control groups for either the HaCaT or A549 cell lines. Even at high concentrations of up to 200 μg/mL, both cancer cells and myoblasts exhibited a survival rate of over 90% after 24 h of incubation with the NIR-CDs (Figure 6a,b). These results provide compelling evidence regarding the excellent biocompatibility and low cytotoxicity of the NIR-CDs [55].

The in vitro fluorescence imaging was carried out using HaCaT and A549 cells. Under blue (402 nm) excitation, bright red light (NIR-CDs) and blue light (DAPI) were observed, as shown in Figure 6c. These images demonstrate that the NIR-CDs can enter and label living cells. The excitation at 402 nm revealed that the bright red fluorescence is mainly concentrated in the cytoplasmic region. The merge images showed minimal overlap between the NIR-CD and 4′,6-Diamidino-2-phenylindole (DAPI) fluorescence, indicating that only a small portion of the NIR-CDs are located in the cell nucleus [56,57]. To investigate whether the NIR-CDs affect DNA replication and transcription, a comet assay was conducted to study the genotoxicity of the NIR-CDs on HaCaT cells. Compared to the control group (Figure 6d,e), the HaCaT cells treated with 200 µg/mL NIR-CDs showed no significant comet tails, while the positive control group treated with H_2_O_2_ (300 µM) displayed significant tailing. These results indicate that NIR-CDs are not genotoxic to HaCaT cells, suggesting their potential as bioimaging agents [58].

#### 2.2.2. In Vivo Bioimaging and Toxicity

**Figure 7:**
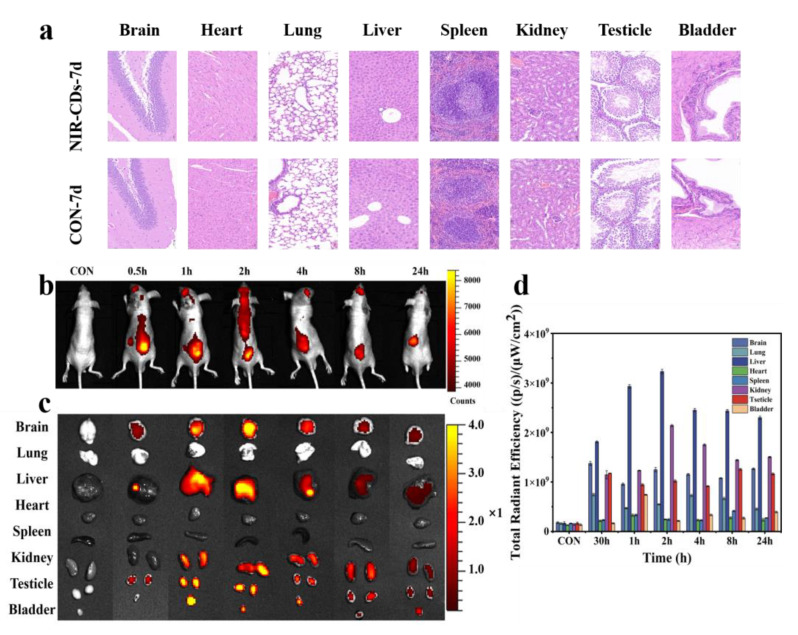
(**a**) H&E (hematoxylin and eosin)-stained tissue slices. (**b**) In vivo imaging of nude mice at different time points. (**c**) Organ imaging in nude mice at different time points. (**d**) Semi-quantitative fluorescence intensity for organ imaging in nude mice at different time points.

The introduction of carbon nanomaterials into the human body may lead to organ damage. Therefore, the in vivo toxicity of the NIR-CDs in various organs (including the brain, heart, liver, lungs, kidneys, spleen, testicles, and bladder) was further assessed in nude mice using histopathological analysis. As shown in Figure 7a, seven days following NIR-CD injection, no histological abnormality was evident in the vital organs, including the heart, brain, spleen, lung, liver, bladder, testis, and kidney, in both the experimental and control groups. These findings suggest that NIR-CDs do not exert any adverse effects on these organs in the short term (7 days), supporting their potential in bioimaging applications. In summary, our preliminary evaluation clearly demonstrated the safety and biocompatibility of NIR-CDs for deep tissue bioimaging [59,60]. These results provide a solid foundation for the further exploration and utilization of NIR-CDs as an effective tool in biomedical research and clinical practice.

To investigate the feasibility of the NIR-CDs for in vivo bioimaging, the imaging performance of NIR-CDs was evaluated in nude mice under 630 nm excitation using an emission wavelength of 670 nm [61]. During the experimental period, no adverse acute toxicological reactions were observed in the mice. A solution of NIR-CDs at a concentration of 0.1 mg/mL in phosphate-buffered saline (PBS) (5% DMSO) (100 µL) was injected subcutaneously into the backs of nude mice. In vivo fluorescence images were acquired at time points of 0.5, 1.0, 2.0, 4.0, 8.0, and 24.0 h (Figure 7b). Bright fluorescence was clearly observed throughout the backs of the mice at the 0.5 and 1.0 h post-injection time points. The fluorescence signals decayed significantly over time, becoming very weak at 24 h post-injection, suggesting that the NIR-CDs had a relatively short residence time in the body and could be rapidly excreted. Fluorescence imaging of the organs was conducted at various time points post-injection, including in the brain, lung, liver, heart, spleen, kidney, testis, and bladder (Figure 7c). As shown in Figure 7d, the fluorescence intensity of these organs exhibited a decline over the course of the observation period. The NIR-CDs accumulated mainly in the brain, liver, kidney, and bladder, with minimal distribution in the lungs, heart, and spleen. Only small amounts of NIR-CDs were found in the liver and brain after 24 h. The NIR-CDs demonstrated the ability to traverse the blood–brain barrier, as evidenced by their capture within the brains of the murine subjects. It is hypothesized that the ultra-small size and the large number of functional groups on the surface may be responsible for the successful crossing. This indicates the potential of NIR-CDs as a new material for the diagnosis and treatment of brain diseases.

Notably, the bladder wall tissue exhibited a more pronounced fluorescence signal due to the absence of urination-related processing. Fluorescence signals in the urine in the bladder were first detected at 1.0 h post-injection and gradually weakened over time, suggesting that NIR-CDs can be rapidly excreted through the renal system. Additionally, imaging of the liver and gallbladder at designated time points revealed intense signals at 1.0 and 2.0 h post-injection, with very low signals observed after 24.0 h, confirming that NIR-CDs are also excreted via bile. These results suggest that subcutaneously injected NIR-CDs rapidly distribute throughout the entire body of mice via the circulatory system rather than accumulating at the injection site [62]. There are two primary metabolic pathways for NIR-CDs, renal excretion and hepatic metabolism, with potential excretion in feces from the duodenum. These findings indicate that the prepared NIR-CDs exhibit good biocompatibility, low biotoxicity, high penetration, and fast excretion in mice. Therefore, NIR-CDs are promising non-toxic nanomaterials for use as diagnostic tools and labeling dyes in biomedical applications.

### 2.3. Antimicrobial Activity

**Figure 8:**
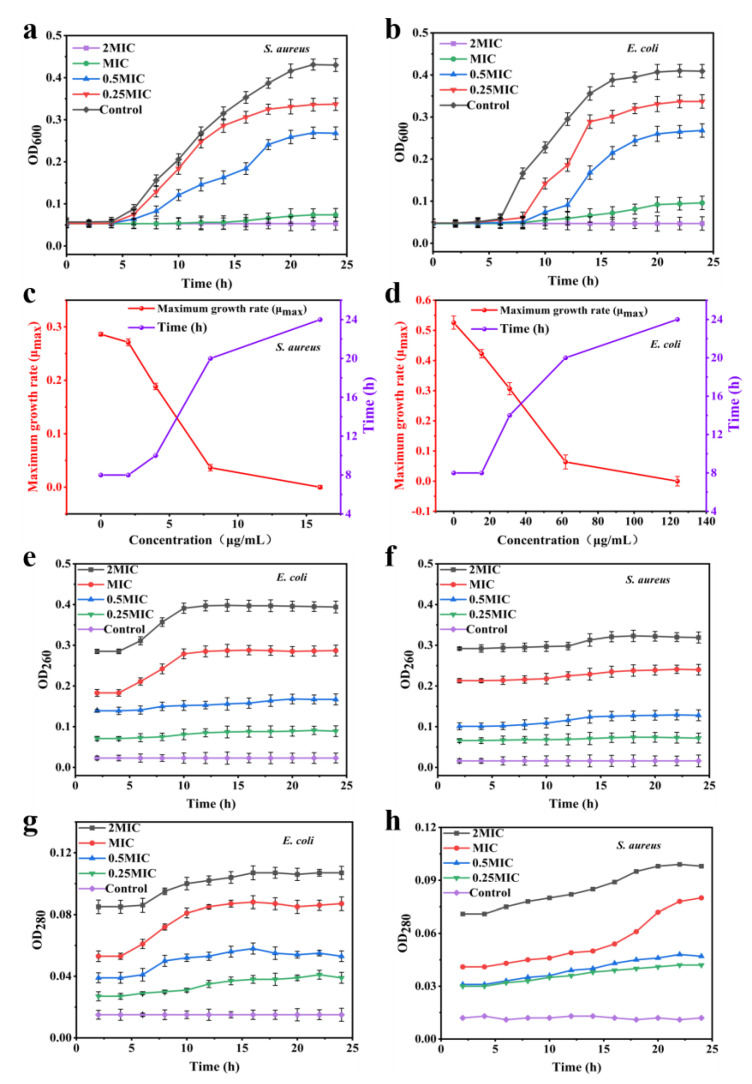
Growth curves of (**a**) *S. aureus* and (**b**) *E. coli* treated with different concentrations of NIR-CDs. The maximum growth rate (μ_max_) and culture time to reach the μ_max_ for (**c**) *S. aureus* and (**d**) *E. coli* after NIR-CD treatment. (**e**–**h**) Effect of NIR-CDs on the ultraviolet absorption of *S. aureus* and *E. coli* at OD_260_ and OD_280_.

In this study, the antimicrobial activities of NIR-CDs against Gram-positive and Gram-negative bacteria were evaluated. The results indicated that the Minimum Inhibitory Concentration (MIC) of NIR-CDs was 8 μg/mL and the Minimum Bactericidal Concentration (MBC) was 32 μg/mL against *S. aureus*. For *E. coli*, the MIC was 62 μg/mL and the MBC was 124 μg/mL. As shown in Figure 8a,b, the control group strains maintained a normal “S”-shaped growth curve. Different concentrations of NIR-CDs significantly inhibited the growth of *S. aureus* and *E. coli*, extending their lag phase. At 2 MIC, almost no bacterial growth was observed. The red line in Figure 8c shows the maximum growth rate (μ_max_) of *S. aureus* after treatment with NIR-CDs. The μ_max_ of the control group was 0.29 h^−1^ after 8 h of incubation. In contrast, the μ_max_ of *S. aureus* after 8, 10, 20, and 24 h of incubation was significantly lower at NIR-CD concentrations of 2, 4, 8, and 16 μg/mL, at 0.27, 0.19, 0.04, and 0 h^−1^, respectively. The blue line in Figure 8c demonstrates that after the NIR-CD treatment, *S. aureus* had μ_max_ values of 0.27, 0.19, 0.04, and 0 h^−1^. This indicates that the bacterial cell cycle was significantly prolonged and growth was markedly delayed. The red line in Figure 8d shows the maximum growth rate (μ_max_) of *E. coli* after NIR-CD treatment. The μ_max_ of the control group was 0.52 h^−1^ after 8 h of incubation. In contrast, the μ_max_ of *E. coli* after 8, 14, 20, and 24 h of incubation was significantly lower at NIR-CD concentrations of 15.5, 31, 62, and 124 μg/mL, with μ_max_ values of 0.42, 0.31, 0.06, and 0 h^−1^, respectively. The blue line in Figure 8d demonstrates that the NIR-CD-treated *E. coli* reached these μ_max_ values, indicating a significantly prolonged bacterial cell cycle and delayed growth. Several studies have reported nitrogen-containing CQDs with assured antimicrobial activity against Gram +ve and Gram −ve microorganisms. Table S1 shows a comparison of the antibacterial activity of the synthesized NIR-CDs from the current investigation to those described in other published work. The Minimum Inhibitory Concentration is often recognized as the gold standard for evaluating a material’s antibacterial properties. Interestingly, in the present study, the NIR-CDs showed greater inhibition of *Staphylococcus aureus* compared to that of previously reported materials. In summary, the NIR-CDs exhibited substantial bacteriostatic effects on both *S. aureus* and *E. coli*.

The cell membrane is a crucial structural component of bacteria, and when it is damaged, nucleic acids, proteins, and other contents leak out [63]. Therefore, the leakage of intracellular substances is a key indicator for evaluating the integrity of the cell membrane. Nucleic acids and proteins within the bacterium exhibit strong absorption peaks at 260 nm and 280 nm. The integrity of the bacterial cell membrane can be assessed by monitoring changes in the absorbance values of these substances in the culture medium. As shown in Figure 8e–h, the absorbance values of the supernatants from the two bacterial cultures not treated with NIR-CDs did not change significantly. In contrast, the absorbance values at 260 nm and 280 nm of the supernatants from the cultures treated with NIR-CDs increased significantly. This indicates that NIR-CDs are destructive to the cell membranes of *S. aureus* and *E. coli*. Moreover, as the concentration of NIR-CDs increased, the degree of cell membrane damage also increased. This demonstrates that, after treatment with NIR-CDs, the integrity of the cell membrane of *S. aureus* and *E. coli* was compromised, leading to the leakage of macromolecules such as nucleic acids and proteins into the extracellular space, thereby exerting an inhibitory effect [64,65,66,67,68].

## 3. Materials and Methods

### 3.1. Chemicals and Instruments

The aerial parts of *Epigynum auritum* were collected in April 2019 from Pu’er City, Yunnan Province, China, and authenticated by Dr. Y.P. Liu from the Kunming Institute of Botany, CAS. A voucher specimen (No. cheng20190415-01) has been deposited in the Faculty of Agriculture and Food at Kunming University of Science and Technology. The HaCaT and A549 cell lines were obtained from the Cell Bank of the Shanghai Institute of Biochemistry and Cell Biology, CAS. Phosphate-buffered saline (PBS), fetal bovine serum (FBS), penicillin, streptomycin, and Roswell Park Memorial Institute (RPMI) 1640 medium were sourced from Gibco (Grand Island, NY, USA). The *S. aureus* and *E. coli* strains were purchased from Shanghai Luwei Technology Co., Ltd., Shanghai, China.

### 3.2. Synthesis of the NIR-CDs

The branches and leaves of *Epigynum auritum* were dried at room temperature. The dried branches and leaves were then finely ground using a micromill. A sample of the powder (10.0 g) was suspended in 200 mL of acetone and subjected to ultrasonic extraction for 30 min. The resulting suspension was then transferred to a Teflon-lined autoclave (500 mL) comprising a poly (tetrafluoroethylene) inner lining, and subjected to a heating process at 120 °C for a period of five hours. The mixture was centrifuged at 8000 rpm for 10 min to remove the supernatant after cooling to room temperature. The supernatant solution was collected, and the dark green supernatant was further filtered through a 0.22 µm polyethersulfone membrane, with the objective of eliminating larger particles. The crude products were subjected to purification via dry silica column chromatography, employing a mixture of ethyl acetate and petroleum ether as the eluent, with the aim of improving the efficiency of separation. Following solvent removal by vacuum drying, the black viscous product, designated as NIR-CDs, was obtained with a 1% yield.

### 3.3. Characterization

The morphology of the NIR-CDs was characterized using transmission electron microscopy (FEI Talos F200x). The NIR-CDs was dispersed in acetone solution, and the liquid was added dropwise to an ultrathin molybdenum mesh. The acetone solution was then allowed to dry naturally. Magnifications of 150 kx and 630 kx were selected for the observation and photographic documentation of the subject. The fluorescence and UV–Vis absorption spectra were collected with an Edinburgh FLS1000 spectrophotometer and a Beijing-Puxi T9CS UV–Vis spectrophotometer. FTIR spectra were recorded using a Perkin-Elmer Spectrum 100 spectrometer, while DLS measurements were conducted with a Zetasizer Nano-ZS90 (Malvern Instruments, Malvern, UK). TGA to assess decomposition temperature was performed on a Netzsch STA449F5 analyzer under nitrogen, from ambient temperature to 780 °C at a heating rate of 10 °C/min. XRD spectra were recorded with Bruker-AXS X-ray diffractometer. The glass transition temperature was analyzed using a DSC2-01180 analyzer. The confocal fluorescence images were taken with a Nikon TI-E-A1R fluorescence microscope with a CCD camera. XPS measurements were performed on an ESCALAB 250 spectrometer. NMR spectra were obtained on a Bruker AVANCE NMR spectrometer (600 MHz) with C_3_D_6_O as the solvent. Photoluminescence (PL) lifetimes were collected on FLS1000. Absolute quantum yield (QY) was measured on FLS 1000 with an integrating sphere (Edinburgh, UK). Cytotoxicity studies were determined using a SpectraMax M5 microplate reader. In vivo imaging was performed with an IVIS Lumina LT system.

### 3.4. Cell Culture

The HaCaT and A549 cell lines were cultured in DMEM (high glucose) supplemented with 10% FBS, 100 μg/mL streptomycin, and 100 U/mL penicillin, and incubated at 37 °C in a 5% CO_2_ incubator.

### 3.5. In Vitro Cytotoxicity Assay

Cytotoxicity against A549 and HaCaT cells was assessed using the CCK-8 assay. Cells (1 × 10^5^ cells/mL) were plated in 96-well plates and incubated in DMEM under a humidified atmosphere with 5% CO_2_ at 37 °C for 24 h. These cells were then incubated with NIR-CDs of different concentrations (0, 12.5, 25, 50, 100, or 200 μg/mL) for another 24 h, respectively. The medium containing NIR-CDs was discarded and the cells were rinsed three times with PBS. The culture medium containing 10% CCK-8 solution was added to each well, and then the 96-cell plate was incubated at 37 °C for another 4 h. The absorbance was determined with a microplate reader (Spectra Max M5, Molecular Devices, Sunnyvale, CA, USA) at 450 nm. The cell survival rate was calculated as

Cell viability (%)=(Atreatment/Acontrol)×100%

Cell viability was expressed as the percentage of OD values compared to the control samples without NIR-CDs. Experiments were performed in triplicates, with six replicate wells for each sample per assay.

### 3.6. Cell Imaging

Both the A549 cells and HaCaT cells at a concentration of 2 × 10^4^ cells/mL were inoculated into the 35 mm diameter laser confocal Petri dishes and cultured in an incubator with 5% CO_2_ at 37 °C. When the cells reached the logarithmic growth phase in the complete medium, NIR-CDs were added into the culture system to achieve a final concentration of 100 μg/mL. After 24 h of incubation, the supernatant was removed and the cells were washed twice with pre-cooled PBS. Then, 1 mL of 4% paraformaldehyde solution was used to fix the cell morphology for 15 min. Following fixation, 1 mL of DAPI solution at a concentration of 0.5 μg/mL was used for 15 min at room temperature and protected from light. The paraformaldehyde solution was removed and the cells were washed twice with pre-cooled PBS. The cells were kept in PBS for bioimaging. Fluorescence images were taken with a confocal laser scanning microscope (CLSM, NIKON TI-E-A1R).

### 3.7. Comet Assay

The comet assay was conducted using a Comet Assay Kit (SCGE) (cat. no. K231211, KeyGEN BioTech, Nanjing, China) following the manufacturer’s protocol. In brief, cells were embedded in 0.7% low-melting-point agarose gel on slides, lysed for 2 h, and incubated in alkaline electrophoresis buffer for 30 min. Electrophoresis was then carried out at 25 V for 30 min. Subsequently, the cells were neutralized, stained with propidium iodide, and imaged using fluorescence microscopy. For each sample, 50 cells were randomly selected for DNA migration length quantification, which was conducted using the Comet Assay Software Project Laboratory (RRID:SCR_007249).

### 3.8. In Vivo Bioimaging and Toxicity Assessment

Male BALB/c nude mice were obtained from Beijing Huafukang Bio-technology Co., Ltd. (certificate number: SCXK (Jing) 2019-0008). All the experimental animal protocols were approved by the Animal Experimental Ethics Committee of Kunming University of Science and Technology. Male nude mice (BALB/c) were 6 weeks of age, weighed 18–22 g, and acclimatized for 7 days after arrival. The imaging performance of NIR-CDs in nude mice was evaluated with an excitation wavelength of 630 nm and an emission wavelength of 670 nm. Male BALB/c nude mice were used for in vivo imaging. Twenty-one mice were divided into 7 groups (*n* = 3). Mice were anesthetized with isoflurane before subcutaneous injection of NIR-CDs. After injection of NIR-CDs (dose: 100 μg/mL), mice were anesthetized at time points of 0.5, 1.0, 2.0, 4.0, 8.0, and 24 h with isoflurane. Then, the imaging of whole-body distribution of NIR-CDs was performed via an IVIS Lumina Series III system (PerkinElmer, Waltham, MA, USA) followed by dissected organ imaging. The data analysis was conducted using Living Image^®^ 4.4 software, with the settings maintained at the standardized level to ensure consistency across the results. No animal showed any signs of acute toxicological reactions during the experimental period. To further evaluate whether the NIR-CDs can cause in vivo toxicity, brain, lung, liver, heart, spleen, kidney, testicle, and bladder samples were collected from the control group and the NIR-CD treatment group one and seven days after subcutaneous injection. Subsequently, the samples were fixed in 4% paraformaldehyde buffer solution for a period of approximately 16 h. The tissue samples were embedded in paraffin blocks and sectioned at a thickness of 5 mm, after which they were subjected to hematoxylin and eosin (H&E) staining.

### 3.9. Antibacterial Activity of NIR-CDs

#### 3.9.1. Determination of Minimum Inhibitory Concentration (MIC) and Minimum Bactericidal Concentration (MBC)

The MIC of NIR-CDs against *S. aureus* and *E. coli* was determined by the two-fold dilution method. Initially, the NIR-CDs were dissolved in DMSO and added to the LB liquid medium to obtain a culture solution containing 500 μg/mL NIR-CDs. This solution was then diluted to different concentration gradients based on the required concentrations for *S. aureus* (128, 64, 32, 16, 8, and 4 μg/mL) and *E. coli* (248, 124, 62, 31, and 15.5 μg/mL). A loopful of *S. aureus* and *E. coli*, stored at −80 °C, was selected and activated on LB agar plates, which were then incubated overnight at 37 °C. The scraped bacteria were diluted in 0.85% NaCl solution, and a standard suspension (10⁶ CFU/mL) was prepared by passing the solution through a MacDonald’s turbidimeter tube (0.5). The assay was conducted using a 96-well microtiter plate, with 100 μL of bacterial solution and 100 μL of diluted medium containing NIR-CDs added to each well. The plate was incubated at 37 °C for 24 h. Bacterial activity was detected using an enzyme marker at OD_600 nm_, and six replicates were prepared for each sample. The concentration at which no bacterial growth was observed was taken as the MIC and expressed as μg/mL.

A volume of 50 μL of the mixture was pipetted from wells with at least the MIC onto LB solid medium, and spread using a spreading stick. After spreading, the medium was incubated at 37 °C for 24 h. Observations were made for any bacterial growth and the colonies were counted. Fewer than 5 colonies indicated the MBC of the near-infrared carbon dots against *S. aureus* and *E. coli*. The experiment was repeated three times for accuracy.

#### 3.9.2. Measurement of the Growth Curve

The log-phase *S. aureus* and *E. coli* were cultured and adjusted to a concentration of 10^8^ CFU/mL with saline. Two milliliters of the bacterial suspension was inoculated into flasks containing 200 mL of sterile LB liquid medium. NIR-CDs (dissolved in DMSO) were added to achieve final concentrations of 16, 8, 4, and 2 μg/mL (*S. aureus*) and 124, 62, 31, and 15.5 μg/mL (*E. coli*). All cultures were incubated on a shaker at 37 °C and 180 rpm. Growth rates and bacterial concentrations were monitored every 2 h by measuring OD_600 nm_ values using a spectrophotometer. The growth rates (*μ*) of *S. aureus* and *E. coli* in the presence of different concentrations of NIR-CDs were calculated using the following formula:

$$\mu =(lnA_{1}-lnA_{2})/(t_{1}-t_{2})$$

where A_1_ and A_2_ are the OD_600 nm_ values at the culture times t_1_ and time t_2_, respectively.

#### 3.9.3. Integrity of Cell Membrane

The NIR-CDs were dissolved in DMSO and added to the liquid medium to achieve final concentrations of 0.25 MIC, 0.5 MIC, MIC, and 2 MIC. Subsequently, a bacterial solution in the growth phase was added to reach a final concentration of 10^7^ CFU/mL. A control group was maintained using a medium containing only DMSO. The samples were incubated for 24 h at 37 °C with continuous oscillation (180 rpm). The bacterial supernatant was collected every 2 h. After incubation, the absorbance of the bacterial supernatant was measured at 260 nm and 280 nm using an enzyme marker. Higher absorbance values indicated more severe damage to the cell membrane.

### 3.10. Statistical Analysis

The significance of the experimental data was analyzed using one-way ANOVA with Tukey’s test, with the software SPSS 19.0 (SPSS Inc., Chicago, IL, USA) employed for this purpose. All figures were plotted using Origin 8.5 software (OriginLab Corporation, Northampton, MA, USA).

## 4. Conclusions

In conclusion, this study demonstrates the successful preparation of NIR-CDs with bioimaging properties and antimicrobial activity derived from the branches and leaves of *Epigynum auritum*. The NIR-CDs exhibit a narrow half-width at half-maximum (FWHM), ultra-small size, amorphous structure, abundant surface groups, low toxicity, and good biocompatibility. In vitro studies confirmed their excellent biocompatibility and low toxicity. The in vivo studies demonstrated their excellent penetration, rapid excretion, and low biotoxicity. The primary metabolic pathways for the NIR-CDs involve renal and hepatobiliary excretion, indicating their potential as bioimaging agents for cellular and in vivo applications. Additionally, the NIR-CDs showed significant inhibitory effects against both Gram-positive bacteria (*S. aureus*) and Gram-negative bacteria (*E. coli*), disrupting the bacterial cell membrane integrity and causing the leakage of intracellular contents. Therefore, this study elucidates the effectiveness of NIR-CDs in antimicrobial therapy and fluorescence imaging diagnosis.

## Data Availability

The data presented in this study are available on request from the corresponding author.

## Supplementary Materials

The following supporting information can be downloaded at: https://www.mdpi.com/article/10.3390/molecules30020422/s1, Figure S1. The absolute PLQY of NIR-CDs in acetone solution under the excitation wavelength of 409 nm. Figure S2. Time-resolved decay spectrum of the NIR-CDs. Figure S3. 1H NMR spectra of the NIR-CDs. Table S1. Comparison of antibacterial activity attained in the present work with that of some other quantum dots. See refs [64,65,66,67,68].
