# Supplementary material for: Epigynum auritum-Derived Near-Infrared Carbon Dots for Bioimaging and Antimicrobial Applications
Source: Molecules. 2025 Jan 20;30(2):422. doi: 10.3390/molecules30020422 (PMC11767839; doi:10.3390/molecules30020422)
Supplement: Supplementary file 1 [file molecules-30-00422-s001.zip › molecules-3357990-supplementary.pdf]

Supporting Information for

Epigynum auritum-Derived Near-Infrared Carbon Dots for Bioimaging and Antimicrobial Applications

Wenfeng Shi [1], Jiahui Li [1], Junmei Pu [1], Guiguang Cheng [1,2], Yaping Liu [1,2], Shanshan Xiao [1,2,]* and Jianxin Cao [1,2,]*

[1] Faculty of Food Science and Engineering, Kunming University of Science and Technology,
Kunming 650500, China; wfshi@stu.kust.edu.cn (W.S.); 20222225041@stu.kust.edu.cn (J.L.); 20222125014@stu.kust.edu.cn (J.P.); ggcheng@kust.edu.cn (G.C.); liuyaping@kust.edu.cn (Y.L.)

[2] Yunnan International Joint Laboratory of Green Food Processing, Kunming 650500, China

*Correspondence: shine330@kust.edu.cn (S.X.); 13313130@kust.edu.cn (J.C.)

The following are included as supporting information for this paper:

Total 4 Pages, 3 Figures.

Figures S1–S3:

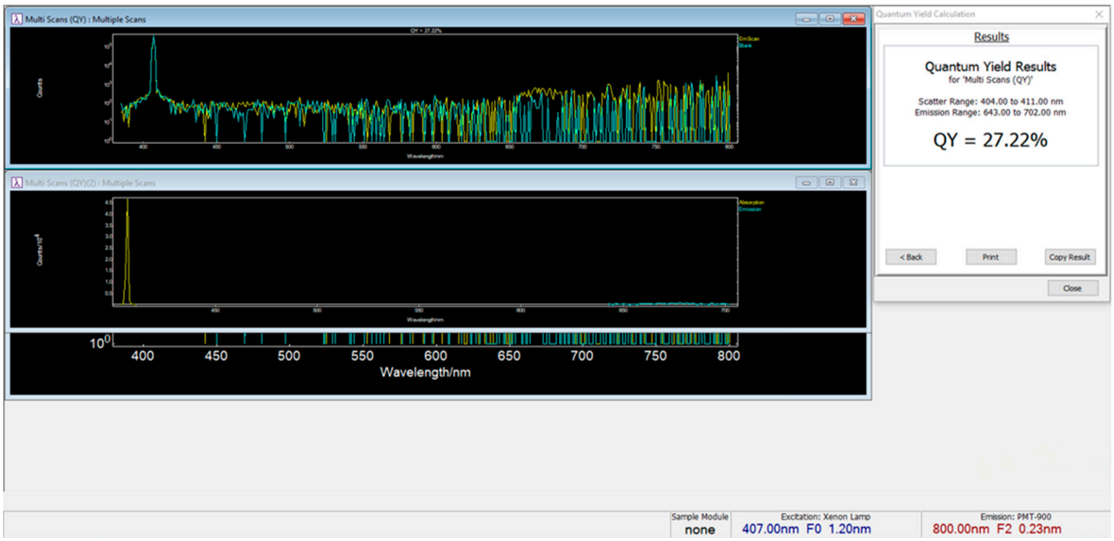

Figure S1. The absolute PLQY of NIR-CDs in acetone solution under the excitation wavelength of 409 nm.

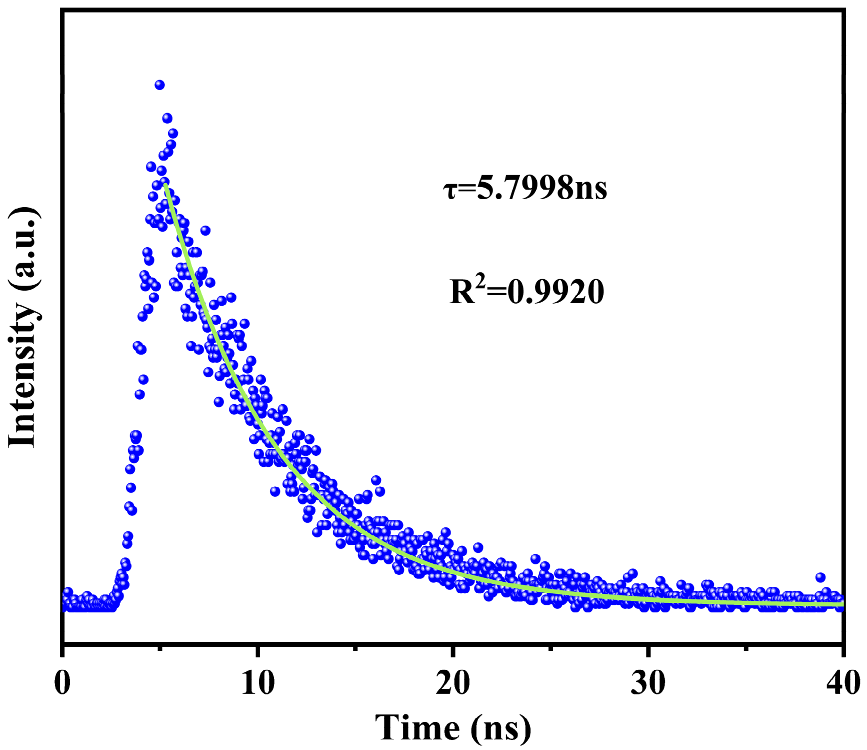

Figure S2. Time-resolved decay spectrum of the NIR-CDs.

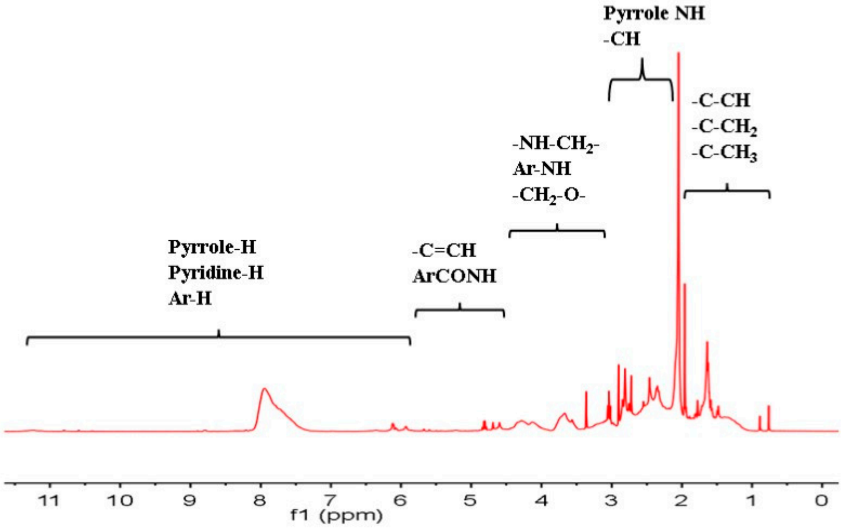

Figure S3. ^{1}H NMR spectra of of the NIR-CDs.

Table S1. Comparison of antibacterial activity attained in the present work with that of some other quantum dots.

Samples	Bacterial species	MIC (μg/mL)	Reference
N、Zn-CDs	*E. coli*	1000μg/mL	[1]
Epigynum auritum-NIR-CDs	*E. coli*	62μg/mL	This Work
HCDs	*E. coli*	20μg/mL	[2]
F-CDs	*E. coli*	64μg/mL	[3]
Lys-CQDs	*E. coli*	62.5μg/mL	[4]
F-CDs	*S. aureus*	128μg/mL	[3]
Curcumin QDs	*S. aureus*	15.6μg/mL	[5]
Lys-CQDs	*S. aureus*	31.25μg/mL	[4]
Epigynum auritum-NIR-CDs	*S. aureus*	8μg/mL	This Work

1. Das, P.; Ganguly, S.; Bose, M.; Mondal, S.; Choudhary, S.; Gangopadhyay, S.; Das, A. K.; Banerjee, S.; Das, N. C., Zinc and nitrogen ornamented bluish white luminescent carbon dots for engrossing bacteriostatic activity and Fenton based bio-sensor. *Materials Science and Engineering: C* **2018**, 88, 115-129.
2. Razavi, R.; Tajik, H.; Molaei, R.; McClements, D. J.; Moradi, M., Janus nanoparticles synthesized from hydrophobic carbon dots and carboxymethyl cellulose: Novel antimicrobial additives for fresh food applications. *Food Bioscience* **2024**, 62, 105171.
3. Liang, J.; Li, W.; Chen, J.; Huang, X.; Liu, Y.; Zhang, X.; Shu, W.; Lei, B.; Zhang, H., Antibacterial Activity and Synergetic Mechanism of Carbon Dots against Gram-Positive and -Negative Bacteria. *ACS Applied Bio Materials* **2021**, 4, (9), 6937-6945.
4. Li, P.; Han, F.; Cao, W.; Zhang, G.; Li, J.; Zhou, J.; Gong, X.; Turnbull, G.; Shu, W.; Xia, L.; Fang, B.; Xing, X.; Li, B., Carbon quantum dots derived from lysine and arginine simultaneously scavenge bacteria and promote tissue repair. *Applied Materials Today* **2020**, 19, 100601.
5. Leong, C. R.; Tong, W. Y.; Tan, W.-N.; Tumin, N. D.; Yusof, F. A. M.; Yacob, L. S.; Rosli, M. I. H. b.; Md Abu, T., Synthesis of curcumin quantum dots and their antimicrobial activity on necrotizing fasciitis causing bacteria. *Materials Today: Proceedings* **2020**, 31, 31-35.
